# Mode of conception in relation to nausea and vomiting of pregnancy: a nested matched cohort study in Sweden

**DOI:** 10.1038/s41598-021-88575-z

**Published:** 2021-04-27

**Authors:** Farnaz Bazargani, S. I. Iliadis, E. Elenis

**Affiliations:** 1 Department of Women’s and Children’s Health, Uppsala University, Uppsala, Sweden; 2 Reproduction Centre, Women’s Clinic, Uppsala University Hospital, 751 85 Uppsala, Sweden

**Keywords:** Diseases, Pathogenesis, Risk factors

## Abstract

Nausea and vomiting of pregnancy (NVP) is a common condition reported however inconclusively among pregnancies after assisted conception. The study objective was thus to explore whether NVP is associated to mode of conception or other in vitro fertilization (IVF)-related variables. This nested matched cohort study, originating from the BASIC-project, was conducted at the Uppsala University Hospital in Sweden between 2010 and 2016. IVF pregnancies (n = 210) and age and parity-matched women with spontaneous pregnancies (n = 420) comprised the study sample. The study outcome was self-reported NVP at gestational week 17. IVF treatment and pregnancy data were obtained after scrutinization of the medical records. NVP with or without medications was not associated with mode of conception (chi-square test, *p* = 0.889), even after adjusting for potential confounders. In a subgroup analysis among IVF pregnancies, NVP without medication was more frequently seen in the group who received cleavage stage embryos *vs* blastocysts (chi-square test, *p* = 0.019), exhibiting a marginally significant but strongly increased effect even after adjustment [crude RRR 3.82 (95% CI 1.23–11.92) and adjusted RRR 3.42 (95% CI 0.96–12.11)]. No difference in the rate of NVP with or without medication between women that underwent fresh and frozen/thawed embryo transfers as well as IVF or ICSI was observed. Conception through IVF is not associated with NVP. Transfer of a blastocyst may decrease the risk of developing NVP and further, large-scale prospective studies are required to validate this finding.

## Introduction

Nausea and vomiting of pregnancy (NVP) is one of the most common conditions during the gestational period^1^. Hyperemesis gravidarum (HG) is the most severe form of NVP, a pregnancy complication characterized by vomiting, excessive loss of body fluid, weight loss above 5% of pre-pregnancy body weight, electrolyte disturbances and ketonuria. HG usually emerges in 0.3–3.6% of all pregnancies^1^, whereas NVP may affect as much as 35–91% of pregnant women^1^ and may have severe consequences on maternal and offspring well-being as well as the healthcare system^2–5^. Although the exact cause of NVP and HG remains unknown, several hypotheses have been proposed indicating a multifactorial etiology. Some studies have examined the potential involvement of a Helicobacter pylori infection during pregnancy^6^, while others focused on the role of elevated estrogens^7^ and human chorionic gonadotropin (hCG) levels in HG-affected pregnancies^8^; however, study findings still remain controversial. Risk factors associated to increased risk for HG are thought to be prior history of HG^9^, young age at pregnancy, primiparity, carrying a female fetus, black or Asian ethnic origin, parathyroid or thyroid dysfunction, and diabetes mellitus type 1^10^.

Pregnancy-related nausea and vomiting, including hyperemesis gravidarum may be accompanied by maternal physical and psychological distress both on the short but also on the long run. Women who are not able to manage excessive stress during pregnancy, depending on either the pregnancy itself as a stressful condition or anxiety due to other reasons such as earlier trauma, may tend to avoid the intolerable situation, by somatization of distress to gastrointestinal symptoms such as nausea and vomiting^11^. In particular, a higher prevalence of sleep disorders, post-traumatic stress disorder, depression, and anxiety in women suffering from prolonged NVP and HG has been demonstrated^12–15^. On the other side, psychiatric manifestations including higher level of anxiety have been reported among infertile couples compared to fertile couples^16^. Individuals that undergo treatment with assisted reproductive technologies (ART) including IVF are unsurprisingly distressed and depressed due to both infertility and uncertainty about the treatment and its results^17^. In fact, it has been demonstrated that women who underwent IVF-treatment experienced more psychological stress and anxiety compared to women with spontaneous pregnancies^18,19^ as well as greater controlling parenting behaviors and emotional bond to the child^19,20^.

Currently, the studies concerning NVP and HG that include IVF populations are limited and with inconclusive findings. One of these studies evaluated the prevalence of NVP in singleton and twin IVF pregnancies and demonstrated an increased NVP risk in the first trimester of pregnancy among twin but not singleton pregnancies^21^. Two other studies explored maternal characteristics among pregnant women with HG in order to estimate the incidence of the condition and identify related risk factors and reported increased risk for HG in ART conceived pregnancies^22,23^. However, the vast majority of studies included a mixed study population comprising pregnancies conceived after IVF, ovarian stimulation or intrauterine insemination, and focused mostly on HG instead of NVP. The question thus of whether there is an association between IVF and NVP remains unanswered.

The primary aim of the present study was therefore to explore the association between mode of conception, IVF or spontaneous, and NVP in a prospective cohort of pregnant women receiving prenatal care at a Swedish University Hospital. Our secondary aim was to explore whether different IVF modalities including fertilization method, fresh or frozen-thawed embryo transferred, and stage of transferred embryo differ regarding the occurrence of NVP.

## Materials and methods

### Study population

The study design is that of a nested matched cohort study originating from the BASIC study. The BASIC-study is a population-based longitudinal study investigating biological correlates of mood and anxiety disorders during pregnancy and the postpartum period^24^, conducted at the department of Obstetrics and Gynecology at Uppsala University Hospital in Sweden between 2010 and 2019. For the present sub-study, approximately 3200 pregnancies were included between January 2010 and September 2016.

All women attending the routine ultrasound examination around gestational week 17 in Uppsala County were invited to participate in the project. Upon invitation, written and oral information was given, and written consent was obtained from women who chose to participate. A self-administered structured questionnaire containing questions on personal history, sociodemographic data and variables related to pregnancy was filled in upon inclusion. Exclusion criteria were: (1) women whose personal data were protected, (2) women who had pathologic pregnancies diagnosed during the routine ultrasound, (3) women who were unable to communicate adequately in Swedish and (4) women younger than 18 years old. About 22% of pregnant women in Uppsala County participated in the project. For the particular matched cohort study further exclusion criteria comprised twin pregnancies and pregnancies conceived through egg donation.

### Exposure

All participating women in the BASIC cohort were asked upon enrollment whether they had conceived through fertility treatment or not. In case of fertility treatment, further information was collected by scrutinizing the participants’ medical records. In total, 210 women who had undergone IVF/ICSI (intracytoplasmic sperm injection) treatment preceding the index pregnancy comprised the exposed group. Two, for each case, age and parity-matched women with spontaneously conceived pregnancies without history of infertility, were randomly selected from the BASIC cohort and comprised the non-exposed group (n = 420).

### Outcome

The main outcome of the study, NVP at GW17, was divided into three groups: (1) absence of NVP, (2) NVP without medication, and (3) NVP with medication. To assess NVP severity, use of antiemetic drugs was utilized as a proxy. NVP was assessed by asking women whether they experienced any kind of nausea in the index pregnancy at GW 17; “Have you experienced any nausea during this pregnancy?” (yes/no), and “Did you receive any medication against nausea of pregnancy?” (yes/no).

### Covariates

Information about sociodemographic and pregnancy related characteristics was gathered upon inclusion through questionnaires; age at delivery (years), body mass index (BMI) before pregnancy (kg/m^2^), parity (primiparity/multiparity), education level (university/secondary school or lower), employment status (full-time or part-time job, studies/maternal leave, sick leave/unemployment), smoking before pregnancy (no/yes), alcohol consumption three months before pregnancy (never/at least once a week/more than once a week), chewing tobacco right before pregnancy (no/yes), depression history (previous depression/contact with psychiatrist or not), delivery fear (no/yes), comorbidities (i.e. migraine, hypertension, diabetes, metabolic disease, allergy, irritable bowel syndrome, alcohol/drug addiction, chronic pain or other disease), and gender of the newborn (male/female). IVF-treatment related information was also recorded such as infertility diagnosis (unexplained/male factor/tubal factor/anovulation/endometriosis/other), male infertility (no/yes), method of conception (IVF/ICSI/combined IVF and ICSI), fresh/frozen-thawed treatments, stage of embryo transferred (cleavage stage/blastocyst), main medicine during IVF treatment (recombinant-FSH or human menopausal used in fresh cycles/hormone replacement therapy or unstimulated cycle used in frozen thawed cycles), ovarian hyperstimulation syndrome (OHSS) in fresh IVF cycles (no/yes), sperm donation (no/yes), number of prior IVF stimulations (1, 2, 3 or more), number of prior embryo transfers (1, 2, 3 or more), number of embryos simultaneously transferred (single/double embryo transfer).

### Statistics

Analyses were carried out with the Statistical Package for the Social Sciences (IBM SPSS Statistics, version 26) and the level of statistical significance was set at 0.05. Maternal characteristics were first tested for normality. Univariate differences in sociodemographic data between exposed and non-exposed group were examined by using chi-square test for categorical variables and the Mann–Whitney U-test for continuous variables. To assess differences in sociodemographic and pregnancy-related data between NVP groups, in the entire study population as well as in the exposed group only, the chi-square test was utilized for categorical variables and the Kruskal–Wallis test for continuous variables. Furthermore, NVP and IVF-modalities were further explored in a subgroup analysis comprising only the exposed group. A multinomial logistic regression analysis was performed first on the entire study population, with NVP as the dependent variable and mode of conception as the independent variable. Relative Risk Ratios (RRR) and 95% Confidence Intervals (95% CI) before and after adjustment for possible confounders were calculated. Women having conceived spontaneously constituted the reference group. The covariates for adjustment were selected a priori based on background knowledge and were further assessed with a Directed Acyclic Graph (DAG) (Supplementary Figure). We regarded employment status and education level both as equal proxies of the individual socioeconomic status, and therefore chose only one variable to include in the DAG and multinomial logistic regression model (i.e., education level). Furthermore, chewing tobacco was not adjusted for due to the great overlap with smoking and to the high rate of missingness (i.e., 54.8%). As a result, the covariates included in the model were: BMI before pregnancy, education level, smoking before pregnancy, alcohol consumption three months before pregnancy, depression history and comorbidities. Further, in the subgroup of exposed women, the effect of IVF modalities on NVP was explored in multinomial logistic regression analysis; the independent variable studied was selected based on statistically significant results of univariate analyses. The multivariate model included all the covariates described above, as well as age at delivery and parity. Lastly, we performed a sensitivity analysis by excluding treatments performed with the use of donated sperm and explored the exposure groups in relation to the study outcome.

### Ethical approval

The study was approved by the Regional Ethical Review Board in Uppsala (Dnr 2009/171), part of the national Swedish ethical review authority. All research was performed in accordance with the relevant guidelines and regulations. Informed consent was obtained from all the participants.

## Results

Table 1 presents sociodemographic and pregnancy-related characteristics in IVF pregnancies and matched spontaneous conceptions. The median age at delivery of the study population was 34.0 years old and 2/3 of study participants were primipara. Compared to spontaneous pregnancies, women who conceived through IVF reported less frequently alcohol consumption before pregnancy (38.4% vs 48.7%, *p* = 0.032), but more frequently comorbidities (59.1% vs 49.9%, *p* = 0.029). The vast majority of study participants had a university degree (83.9%). No other differences on sociodemographic and clinical characteristics were observed between the two groups.

**Table 1:** Sociodemographic and pregnancy-related characteristics in IVF pregnancies and matched spontaneous conceptions.

		Total (N = 630)	Spontaneous conception (N = 420)	IVF conception (N = 210)	p-value
		**Median (IQR**^d^**)** **Range**			
Age at delivery (years)		630	34.0 (5.0)	34.0 (5.0)	0.999^a^
			23–42	23–42	
BMI before pregnancy (kg/m^2^)		627	22.7 (4.0)	23.3 (5.7)	0.123^a^
			16.0–50.1	17.5–36.4	
		**N (%)**			
Parity	Primipara	420	280 (66.7)	140 (66.7)	0.999^b^
	Multipara	210	140 (33.3)	70 (33.3)	
Education level	University	528	359 (85.7)	169 (80.5)	0.094^b^
	Secondary school or lower	101	60 (14.3)	41 (19.5)	
Employment status	Maternal leave, sick leave, unemployment	579	388 (93.0)	191 (91.0)	0.352^b^
	Full-/part-time job, studies	48	29 (7.0)	19 (9.0)	
Smoking before pregnancy	No	413	273 (65.6)	140 (67.6)	0.618^b^
	Yes	210	143 (34.4)	67 (32.4)	
Alcohol consumption before pregnancy	Rarely/never	152	99 (51.3)	53 (61.6)	0.032^b^
	At least once a week	98	68 (35.2)	30 (34.9)	
	More than once a week	29	26 (13.5)	3 (3.5)	
Chewing tobacco before pregnancy	No	266	183 (92.9)	83 (94.3)	0.656^b^
	Yes	19	14 (7.1)	5 (5.7)	
History of depression/contact with psychiatrist	No	252	175 (41.8)	77 (36.8)	0.236^b^
	Yes	376	244 (58.2)	132 (63.2)	
Delivery fear	No fear	467	316 (78.2)	151 (76.3)	0.589^b^
	Any fear	135	88 (21.8)	47 (23.7)	
Comorbodities^c^	No	294	209 (50.1)	85 (40.9)	0.029^b^
	Yes	331	208 (49.9)	123 (59.1)	
Newborn gender	Female	305	213 (50.8)	92 (43.8)	0.096^b^
	Male	324	206 (49.2)	118 (56.2)	

^a^Mann–Whitney U-test.

^b^Chi-square test.

^c^Comorbidities include migraine, hypertension, diabetes, metabolic disease, allergy, irritable bowel syndrome, alcohol/drug addiction, chronic pain or other disease.

^d^Interquartile range.

Table 2 presents sociodemographic and pregnancy-related characteristics of the study sample by nausea group. NVP, with or without medication, was not associated with mode of conception (*p* = 0.889). A higher percentage of women without NVP were primipara compared to women with NVP either requiring medication or not (80.5% vs. 60.9% vs. 62.3%, *p* < 0.001). A higher proportion of women with NVP requiring medication or not reported history of depression compared to women in the non-NVP group (69.6% and 63.4% vs. 46.2%, *p* < 0.001). Lastly, a lower percentage of women with NVP not requiring medication reported smoking before pregnancy in comparison to the other outcome groups. After performing a multinomial logistic regression analysis, it was confirmed that the association between mode of conception and NVP severity was not statistically significant; women conceiving through IVF exhibited a crude relative risk ratio (RRR) of 0.92 (95% CI 0.63–1.36) for NVP without medication and a crude RRR of 1.01 (95% CI 0.56–1.83) for NVP with medication, relative to spontaneously conceiving women. The associations remained unchanged even after adjustment for various covariates (i.e., the likelihood of suffering from NVP without medication and NVP with medication among women conceiving with IVF were adjusted RRR 0.69 (95% CI 0.37–1.29) and adjusted RRR 0.78 (95% CI 0.28–2.16), respectively, compared to women conceiving spontaneously given that the other covariates were held constant).

**Table 2:** Sociodemographic and pregnancy-related characteristics of study sample by nausea group.

		Total	No NVP (N = 159)	NVP without medication (N = 400)	NVP with medication (N = 69)	p-value
			**Median (IQR**^d^**)** **Range**			
Age at delivery (years)		628	34.0 (5.0)	34.0 (6.0)	35.0 (4.0)	0.540^a^
			23–42	23–42	25–41	
BMI before pregnancy (kg/m^2^)		625	23.4 (4.3)	22.7 (3.9)	23.1 (7.0)	0.208^a^
			16.0–50.9	17.5–39.0	18.0–39.8	
			**N (%)**			
Mode of conception	Spontaneous	418	104 (65.4)	269 (67.3)	45 (65.2)	0.889^b^^,f^
	IVF	210	55 (34.6)	131 (32.7)	24 (34.8)	
Parity	Primipara	419	128 (80.5)	249 (62.2)	42 (60.9)	< 0.001^b^
	Multipara	209	31 (19.5)	151 (37.8)	27 (39.1)	
Education level	University	526	127 (79.9)	340 (85.2)	59 (85.5)	0.280^b^
	Secondary school or lower	101	32 (20.1)	59 (14.8)	10 (14.5)	
Employment status	Maternal leave, sick leave, unemployment	48	9 (5.7)	32 (8.0)	7 (10.3)	0.440^b^
	Full-/part-time job, studies	577	150 (94.3)	366 (92.0)	61 (89.7)	
Smoking before pregnancy	No	413	96 (60.8)	277 (70.1)	40 (58.8)	0.039^b^
	Yes	208	62 (39.2)	118 (29.9)	28 (41.2)	
Alcohol consumption before pregnancy	Rarely/never	152	36 (50.0)	101 (55.8)	15 (62.5)	0.744^b^
	At least once a week	96	26 (36.1)	63 (34.8)	7 (29.2)	
	More than once a week	29	10 (13.9)	17 (9.4)	2 (8.3)	
Chewing tobacco before pregnancy	No	264	70 (92.1)	172 (94.0)	22 (91.7)	0.813^b^
	Yes	19	6 (7.9)	11 (6.0)	2 (8.3)	
History of depression^e^	No	252	85 (53.8)	146 (36.6)	21 (30.4)	< 0.001^b^
	Yes	374	73 (46.2)	253 (63.4)	48 (69.6)	
Delivery fear	No fear	465	125 (80.1)	293 (77.5)	47 (71.2)	0.347^b^
	Any fear	135	31 (19.9)	85 (22.5)	19 (28.8)	
Comorbodities^c^	No	294	91 (57.6)	172 (43.4)	31 (44.9)	0.010^b^
	Yes	329	67 (42.4)	244 (56.6)	38 (55.1)	
Newborn gender	Female	303	77 (48.4)	193 (48.4)	33 (47.8)	0.998^b^
	Male	324	82 (51.6)	206 (51.6)	36 (52.2)	

^a^Kruskal–Wallis test.

^b^Chi-square test.

^c^Comorbidities include migraine, hypertension, diabetes, metabolic disease, allergy, irritable bowel syndrome, alcohol/drug addiction, chronic pain or other disease.

^d^Interquartile range.

^e^Or contact with psychiatrist.

^f^Not statistically significant association even after multinomial logistic regression analysis, before and after adjustment for BMI before pregnancy, education level, smoking before pregnancy, alcohol consumption 3 months before pregnancy, depression history and comorbidities.

Table 3 presents pregnancy and IVF-treatment characteristics in relation to NVP in the exposed group. We could not find any significant difference in the rate of NVP with or without medication between women that underwent fresh and frozen/thawed embryo transfers or in the prevalence of OHSS in fresh IVF cycles. The same applied when testing differences between NVP groups in relation to fertilization by IVF or ICSI. However, we observed a significant difference between embryo stage and the study outcome; women with NVP non-requiring medication had received more often cleavage stage embryos compared to women without NVP and women with NVP requiring medication (93.5% vs. 78.9% vs. 75.0%, *p* = 0.019). All findings remained unchanged after the sensitivity analysis performed. Lastly, we calculated the effect estimate of embryo stage on NVP. It was estimated that the relative risk for NVP without medication of cleavage stage embryos relative to blastocysts was increased by a factor of 3.82 and 3.42, before and after the adjustment for relevant covariates [i.e., crude RRR 3.82 (95% CI 1.23–11.92) and adjusted RRR 3.42 (95% CI 0.96–12.11) respectively].

**Table 3:** Pregnancy and IVF-treatment characteristics in relation to NVP in the exposed group.

		Total	No NVP (N = 55)	NVP without medication (N = 131)	NVP with medication (N = 24)	p-value
		**Median (IQR**^f^**)** **Range**				
Age at delivery (years)		210	34.0(5.0)	34.0 (5.0)	34.5 (5.0)	0.850^a^
			23–41	25–42	25–39	
BMI before pregnancy (kg/m^2^)		210	22.6 (6.5)	23.2 (4.8)	25.9 (6.6)	0.055^a^
			18.0–35.3	17.5–36.4	19.7–33.5	
			**N%**			
Parity	Primiparity	140	43 (78.2	82 (62.6)	15 (62.5)	0.108^b^
	Multiparity	70	12 (21.8)	49 (37.4)	9 (37.5)	
IVF cycle	Fresh	108	33 (70.2)	61 (57.5)	14 (70)	0.249^b^
	Frozen/thawed	65	14 (29.8)	45 (42.5)	6 (30)	
Conception method	IVF	101	28 (70.0)	66 (68.0)	7 (41.2)	0.150^b^
	ICSI	51	12 (30.0)	29 (29.9)	10 (58.8)	
	Combined	2	0 (0)	2 (2.1)	0 (0)	
Transferred Embryo stage	Cleavage stage	128	30 (78.9)	86 (93.5)	12 (75.0)	0.019^b^
	Blastocyst	18	8 (21.1)	6 (6.5)	4 (25.0)	
Main medicine during IVF stimulation	rFSH^c^	71	20(48.8)	43 (45.3)	10 (62.6)	0.316^b^
	HMG^d^	24	9 (22.0)	13 (13.7)	2 (12.5)	
	HRT^e^	12	2 (4.9)	9 (9.5)	1 (6.3)	
	Unstimulated cycle (frozen)	41	10 (24.4)	29 (30.5)	2 (12.5)	
OHSS in fresh IVF treatment	No	84	21 (77.8)	52 (88.1)	11 (78.6)	0.399^b^
	Yes	16	6 (22.2)	7 (11.9)	3 (21.4)	
Embryos transferred	Single	149	41 (95.3)	91 (95.8)	17 (100)	0.676^b^
	Double	6	2 (4.7)	4 (4.2)	0 (0)	
Number of previous IVF treatments	0	10	2 (4.3)	8 (8.0)	0 (0)	0.645^b^
	1	94	29 (63.0)	53 (53.0)	12 (70.6)	
	2	42	12 (26.1)	27 (27.0)	3 (17.6)	
	3 or more	17	3 (6.5)	12 (12.0)	2 (11.8)	
Number of previous embryo transfers	0	3	2 (4.4)	1 (1.0)	0 (0)	0.656^b^
	1	76	25 (55.69	41 (41.4)	10 (58.8)	
	2	30	5 (11.1)	23 (23.2)	(2 (11.8)	
	3 or more	52	13 (28.9)	34 (34.3)	5 (30.4)	
Sperm donation	No	152	41 (85.4)	93 (89.4)	18 (94.7)	0.529^b^
	Yes	19	7 (14.6)	11 (10.6)	1 (5.3)	
Infertility diagnosis	Unexplained	53	17 (54.8)	33 (38.8)	3 (21.4)	0.587^b^
	Male factor	32	6 (19.4)	21 (24.7)	5 (35.7)	
	Tubal factor	6	1 (3.2)	3 (3.5)	2 (14.3)	
	Anovulation	26	5(16.1)	18 (21.2)	3 (21.4)	
	Endometriosis	12	2 (6.5)	9 (10.6)	1 (7.1)	
	Other	1	0 (0)	1 (1.2)	0 (0)	
Male infertility	No	134	37 (75.5)	84 (72.4)	13 (61.9)	0.504^b^
	Yes	52	12 (24.5)	32 (27.6)	8 (38.1)	

^a^Kruskal–Wallis test.

^b^Chi-square test.

^c^Recombinant FSH: Follitropin alfa, Follitropin beta.

^d^HMG (Human menopausal gonadotropin).

^e^HRT (Hormone Replacement Therapy): Progynon (Estradiol) + Lutinus (Progesterone) or Crinone. (Progesterone).

^f^Interquartile range.

## Discussion

### Main findings and comparison to other studies

In the present study, no significant association between mode of conception and NVP could be demonstrated. Furthermore, NVP was not associated to different IVF modalities such as fertilization method, stimulation protocol, or use of cryopreservation. On the contrary, the developmental stage of the embryo transferred was associated with the occurrence of NVP since NVP without medication was more often present in pregnancies after cleavage stage embryo transfer.

Similar findings were reported in the study by Bordi et al.^25^ who assessed the effects of ART on maternal and perinatal outcomes in twin pregnancies. In this study, no significant difference in the prevalence of HG between ART and spontaneous pregnancies was observed. However, this study had HG and not NVP as main study outcome. On the contrary, Roseboom et al.^23^, demonstrated a higher rate of ART conceived pregnancies among women suffering from HG, while Nurmi et al.^22^, found an increased incidence of HG among ART conceived pregnancies. However, both studies comprised ART pregnancies which included IVF and other assisted reproductive techniques besides IVF, while only IVF-conceived pregnancies were included in our study. Subsequently, a mixed population could affect the interpretation of the findings, since it is unclear whether the observed association is due to the impact of infertility or due to the treatment method. Furthermore, our study assessed the whole spectrum of NVP and was not restricted to women with HG. These differences in study design may partly explain the different results observed.

Interestingly, the rate of NVP not requiring medication in our study was higher among pregnancies after cleavage stage embryo transfer compared to pregnancies resulting from blastocyst transfer. In the IVF treated group, information about the stage of embryo transferred was available for 146 women, the majority of whom had received cleavage-stage embryos (n = 128, 87.7%). The rate of blastocyst transfers during that period was lower compared to current trends. It is however in agreement with the global trend during that period. In accordance with our data, national statistics of treatments performed in Sweden in 2012 demonstrated that 87.4% of fresh transfers and 62.7% of frozen transfers were done with embryos in cleavage stage, which have thereafter gradually been replaced by blastocysts, first in frozen-thawed and afterwards even in fresh cycles. In 2016, the percentage of cleavage stage embryos transferred was 74% for fresh and 18% for frozen-thawed cycles^26^. Nevertheless, this finding should be interpreted with caution due to the missing rate of information and the low rate of blastocyst transfers during that time-period.

Mechanisms proposed behind the development of NVP comprise the presence of an on-going gastric H-pylori infection^6^, as well as increased levels of hCG and estradiol^27^. In particular, higher levels of hCG positively correlate with NVP incidence, an association that has been consistently reported in molar and multiple gestations^27^. The hCG-vomiting effect has been attributed to a stimulatory effect of the upper gastrointestinal tract secretions^7^ and a triggering of the nausea and vomiting reflex in the brain stem^28^. The association between hCG and NVP could not however be verified by all studies probably due to variation in either the biologic activity of hCG or the individual susceptibility to the hCG hormone^27^. Concerning estradiol, the mechanism associating it to HG is thought to be mediated by the increased gastric fluid content and slower gastrointestinal transit which may exacerbate nausea and vomiting^7^. In fact, cigarette smoking during pregnancy is associated with lower levels of hCG and estradiol^29^, and it is thus not surprising that a lower rate of self-reported NVP without medications has been observed among smokers in our study.

### Strengths and limitations

The population-based prospective design and the large number of background variables constitute the main strengths of this study. Furthermore, the utilization of two age and parity matched controls for each exposed woman originating from the same cohort is also a strength of the study. We chose to match the two groups for age and parity since both younger age and nulliparity have been consistently reported as risk factors for NVP^10,22,30^. Additionally, only IVF treated women were included in order to increase the specificity of the findings.

The self-reported information regarding the outcome and a number of covariates should be considered as a limitation, along with a higher missing rate on certain IVF-related variables. Moreover, a larger study sample could have contributed to an even better study power, especially when examining IVF characteristics in the exposed group. Since no prior reports existed regarding the occurrence of NVP in IVF treated women, no power calculation could be performed a priori. The latter explains why this study should be treated as an exploratory study in the field. Regarding classification of NVP into subgroups, it should be kept in mind that, despite our attempt to assess disease severity based on antiemetic medication use, other aspects such as psychological factors may influence the woman’s self-perceived symptoms^27^. The higher education of participants in the BASIC project, compared with those residing in Uppsala County, as well as the relatively low participation rate in the BASIC study should be taken into account in terms of generalizability of study results.

## Conclusion

Conception mode was not associated with NVP development in our study. Interestingly, cleavage stage embryo transfer was associated with a higher occurrence of NVP without medication. The developmental stage of the transferred embryo may therefore affect NVP development and should be thus taken into account when planning an IVF treatment cycle, especially among women with prior NVP history. Future research with large-scale studies is needed in order to validate our study findings and further examine the possible effect of transferred embryo stage on NVP.

## Supplementary Information

Supplementary Figure.

## Data Availability

The data that support the findings of this study are available upon reasonable request from the corresponding author. The data are not publicly available due to privacy and ethical restrictions.
