# Supplementary material for: Mode of conception in relation to nausea and vomiting of pregnancy: a nested matched cohort study in Sweden
Source: Sci Rep. 2021 Apr 27;11:9039. doi: 10.1038/s41598-021-88575-z (PMC8079670; doi:10.1038/s41598-021-88575-z)
Supplement: Supplementary file 1 — Supplementary Figure. [file 41598_2021_88575_MOESM1_ESM.pdf]

Mode of conception in relation to nausea and vomiting of pregnancy: a nested matched cohort study in Sweden

F Bazargani[1,2], S I Iliadis[1,2], E Elenis[1,2]

[1] Department of Women's and Children's Health, Uppsala University, Uppsala, Sweden
[2] Reproduction Centre, Women's Clinic, Uppsala University Hospital, Uppsala, Sweden

Corresponding author:

Farnaz Bazargani
Telephone number: (+46) 737108866
Email: farnaz.bazargani@kbh.uu.se
Uppsala University Hospital
SE 751 85 Uppsala, Sweden

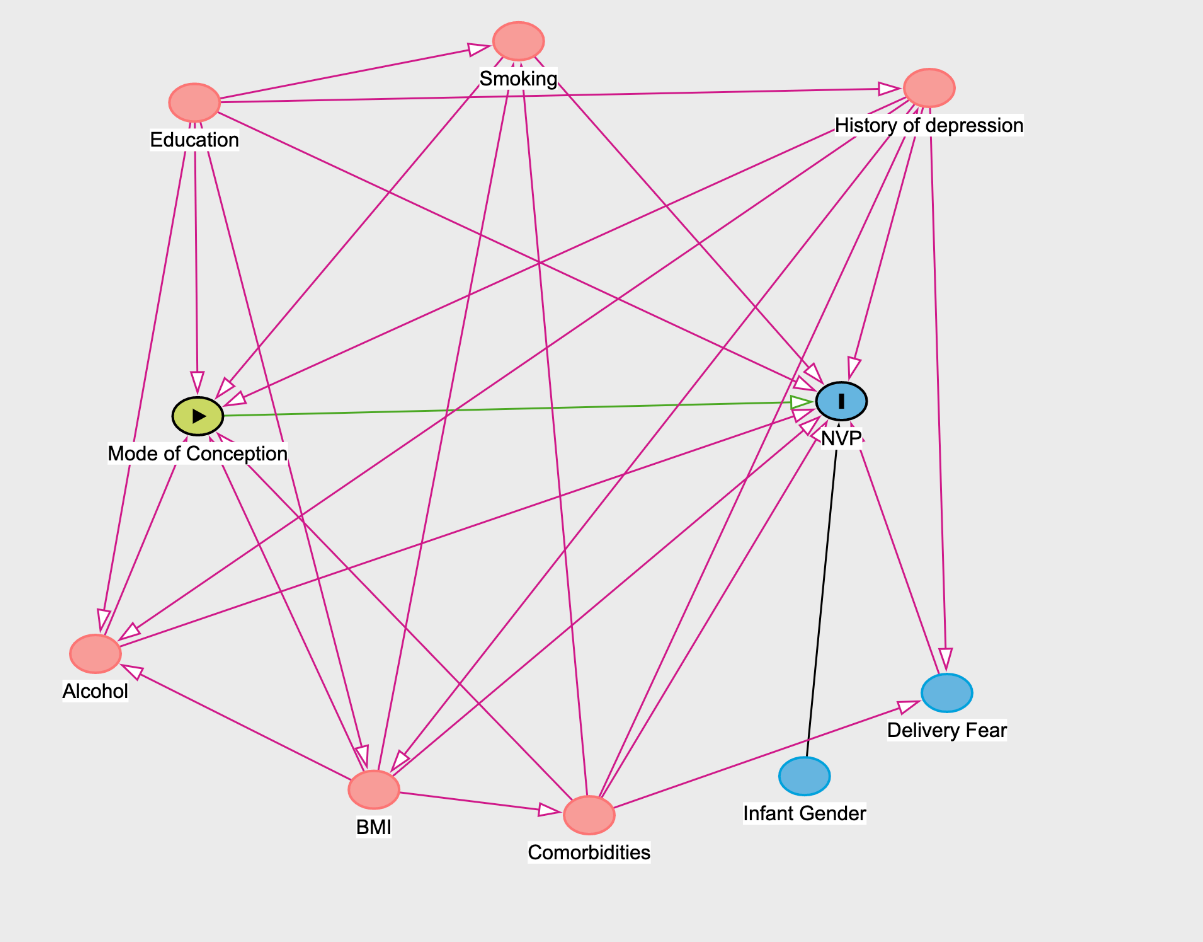
Smoking
Education
History of depression
Mode of Conception
NVP
Alcohol
Delivery Fear
BMI
Infant Gender
Comorbidities
